# Overview of General and Discriminating Markers of Differential Microglia Phenotypes

**DOI:** 10.3389/fncel.2020.00198

**Published:** 2020-08-06

**Authors:** Agnieszka M. Jurga, Martyna Paleczna, Katarzyna Z. Kuter

**Affiliations:** Maj Institute of Pharmacology, Department of Neuropsychopharmacology, Polish Academy of Sciences, Krakow, Poland

**Keywords:** microglial heterogeneity, infiltrating macrophages, inflammation, polarization, M1/M2 phenotype, neurotoxicity, regeneration

## Abstract

Inflammatory processes and microglia activation accompany most of the pathophysiological diseases in the central nervous system. It is proven that glial pathology precedes and even drives the development of multiple neurodegenerative conditions. A growing number of studies point out the importance of microglia in brain development as well as in physiological functioning. These resident brain immune cells are divergent from the peripherally infiltrated macrophages, but their precise *in situ* discrimination is surprisingly difficult. Microglial heterogeneity in the brain is especially visible in their morphology and cell density in particular brain structures but also in the expression of cellular markers. This often determines their role in physiology or pathology of brain functioning. The species differences between rodent and human markers add complexity to the whole picture. Furthermore, due to activation, microglia show a broad spectrum of phenotypes ranging from the pro-inflammatory, potentially cytotoxic M1 to the anti-inflammatory, scavenging, and regenerative M2. A precise distinction of specific phenotypes is nowadays essential to study microglial functions and tissue state in such a quickly changing environment. Due to the overwhelming amount of data on multiple sets of markers that is available for such studies, the choice of appropriate markers is a scientific challenge. This review gathers, classifies, and describes known and recently discovered protein markers expressed by microglial cells in their different phenotypes. The presented microglia markers include qualitative and semi-quantitative, general and specific, surface and intracellular proteins, as well as secreted molecules. The information provided here creates a comprehensive and practical guide through the current knowledge and will facilitate the choosing of proper, more specific markers for detailed studies on microglia and neuroinflammatory mechanisms in various physiological as well as pathological conditions. Both basic research and clinical medicine need clearly described and validated molecular markers of microglia phenotype, which are essential in diagnostics, treatment, and prevention of diseases engaging glia activation.

## Introduction

Why are cells previously considered as merely “brain glue” now gaining such a tremendous amount of interest? Microglia were, for a long time, considered to have potentially deleterious functions, but further studies showed that they can act both in a cytotoxic and in a neuroregenerative way depending on their current phenotype. Central nervous system (CNS) microglial cells show a complex set of phenotypes dynamically changing in physiology and pathology. Therefore, it is essential to precisely recognize their activation states and functions *via* specific markers.

In this review, we will focus on microglial heterogeneity. We will describe its general markers, including those selectively discriminating microglia from peripheral macrophages, as well as indicators of activation and of particular phenotypes. Qualitative markers vs. semi-quantitative expression of different protein ratios will also be shown here. The importance of accurate microglial phenotype description is depicted *via* examples of inflammatory processes in the aging brain, as well as in diseases and their diagnostics. Shifting between microglial phenotypes has recently become a therapeutic approach in multiple CNS diseases. Definite description of microglia profiles is an important challenge in today’s drug design studies. The information gathered here will be helpful in choosing the appropriate descriptors or targets for studies and will allow more precise identification of microglial states in research while defining immunological mechanisms in the brain.

## The Nature of Microglia, Their Role, and Their Function in The Brain

**Figure 1:**
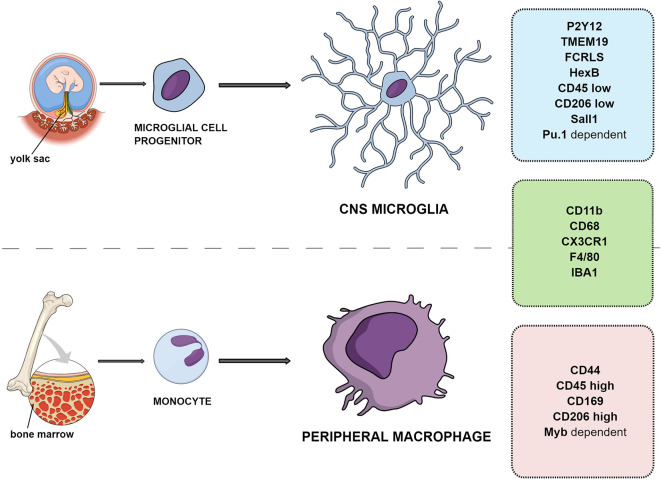
Differences and similarities between resident microglia and peripheral macrophage ontogeny and markers.

Microglial cells are the resident macrophages of CNS and account for about 10% of the cell population, with a mean density of ca. 70 cells/mm^2^ in mouse brain tissue. The estimated number of microglial cells in the CNS reaches 3.5 million (Lawson et al., 1990). The other source of immunocompetent cells in the brain are the macrophages infiltrating from the periphery (Daneman et al., 2010). Ontogenetically, the microglial population differentiates from the embryonic yolk sac, while peripheral macrophages are the monocytes originating from the hematopoietic stem cells and maturating in bone marrow (Gomez Perdiguero et al., 2013; Figure 1). For the purposes of this review, the resident CNS macrophage population will be referred to as microglia and peripherally originating monocytes will be called macrophages. In contrast to neurons, microglial cells have the ability to completely restore their population in the adult brain. According to recent rodent studies, less than 1% of the microglial population was able to completely restore the original density in 1 week (Elmore et al., 2015). The human microglial population renews at a rate of ca. 28% per year, meaning that a microglial cell is approx. 4.2 years old (Réu et al., 2017).

In the developing brain, microglia function to shape and protect the environment. They take part in synaptic remodeling, pruning, vessel patterning, and angiogenesis promotion (Du et al., 2017). The general functions of microglia in the adult brain are to monitor the environment and to start an inflammatory response in case of the detection of any danger signal. They are the first line of defense in the brain. Microglia can recognize pathogens and subtle changes in the microenvironment with surface receptors that detect complement fragments, immunoglobulins, adhesion molecules, chemokine receptors (CCR), Toll-like receptors (TLR), purinoceptors, and scavenger and Fc receptors. When any potential danger is recognized, microglia become activated and communicate by cytokine release, which alerts surrounding cells and influences their functioning. This triggers an inflammatory state, a physiological condition that is strictly regulated and silenced over time.

If neuronal malfunction is detected, microglia are also responsible for introducing cellular death (Wake et al., 2009; Hornik et al., 2016). This may be triggered *via* microglial NMDA receptor activation and consequent inducible nitric oxide synthase (iNOS) upregulation and secretion of multiple inflammatory and cytotoxic factors (Kaindl et al., 2012). When inflammation is very strong or its regulation fails and the pro-inflammatory phase is prolonged, it can be harmful to the tissue environment, and microglia can even kill healthy neurons (Brown and Vilalta, 2015; Gomes-Leal, 2019).

Cellular debris and cytotoxic protein aggregates released by other cells can also be phagocytosed by microglia, cleaning the extracellular space (Neumann et al., 2009; Herzog et al., 2019). Importantly, microglia also have protective functions. They release anti-inflammatory cytokines, silencing the local inflammation, produce mediators of myelin repair, participating in axonal regeneration and neurogenesis, and also promote trophic support by secretion of neurotrophins (Gomes-Leal, 2012).

## General Microglia Markers

When choosing microglia markers for visualization, their localization is of the essence. Microglia markers include surface, intracellular (cytosolic proteins, gene transcripts), and released molecules. The general microglia markers can be detected irrespective of current cell phenotype. The most widely used markers are ionized calcium-binding adapter molecule 1 (IBA-1; Yun et al., 2018), cluster of differentiation receptors (CD68, CD11b, CD14, CD45, CD80, and CD115; Zanoni et al., 2011; Fadini et al., 2013; Jenkins et al., 2013; Jeong et al., 2013; Rice et al., 2017), fractalkine receptor (CX3CR1; Jones et al., 2010), ferritin (Holland et al., 2018), F4/80 (Lin et al., 2005), high-affinity immunoglobulin epsilon receptor subunit gamma (FCER1G) and vimentin (Lebedeva et al., 2005; Mukherjee et al., 2016). Many of those markers can be detected in other cells also, like CD68 in infiltrating macrophages and vimentin in astroglia. To the current knowledge, the most specific general microglia markers are transmembrane protein 119 (TMEM119) and purinergic receptor P2Y12R (Butovsky et al., 2014; Table 1).

**Table 1:** Overview of microglial cell marker proteins, their alternative names, function, and localization.

Marker	Full name	Synonyms	Functions	References
**General microglia markers Membrane proteins**				
*CD11B*	cluster of differentiation 11b	integrin subunit alpha M, ITGAM; complement receptor 3 alpha, CR3A	alpha subunit of an integrin complement receptor part 3 (MAC-1); involved in adhesion processes and uptake of complement-coated molecules	Chakrabarty et al. (2010) and Jeong et al. (2013)
*CD14*	cluster of differentiation 14	myeloid cell-specific leucine-rich glycoprotein, monocyte differentiation antigen CD14	co-receptor for transmembrane TLR4 and endosomal TLR7/9 presenting antigens to them	Baumann et al. (2010) and Zanoni et al. (2011)
*CD16*	cluster of differentiation 16	low-affinity immunoglobulin gamma Fc region receptor III, Fc-gamma RIII	Fc receptor detecting immunoglobulin gamma (IgG) antibodies; engaged in phagocytosis processes	Nagarajan et al. (1995)
*CD40*	cluster of differentiation 40	tumor necrosis factor receptor superfamily member 5	transduces signals activating ERK kinase	Lebedeva et al. (2005)
*CD45*	cluster of differentiation 45	receptor-type tyrosine-protein phosphatase C, PTPRC	includes enzymatic subunit; positive regulator of T-cell activation	Rice et al. (2017)
*CD80*	cluster of differentiation 80	T-lymphocyte activation antigen CD80, Activation B7–1 antigen	together with CD86, CD28, and ICAM1, generates the co-stimulatory signals after MHCII activation	Park et al. (2003) and Lebedeva et al. (2005)
*CD68*	cluster of differentiation 68	macrosialin	strongly upregulated during inflammation; it able to internalize from cell surface to endosomes immediately after stimulation	Holness and Simmons (1993), Kurushima et al. (2000) and Fadini et al. (2013)
*CD115*	cluster of differentiation 115	macrophage colony-stimulating factor 1 receptor, M-CSFR; colony-stimulating factor 1 receptor, CSF-1R	recognizes pro-inflammatory ligands like IL-34 or CSF-1—cytokines controlling proliferation, differentiation and general functioning of macrophages/microglia	Jenkins et al. (2013)
*CX3CR1*	CX3C chemokine receptor 1	fractalkine receptor, C-X3-C CKR-1, Beta chemokine receptor-like 1, CMK-BRL-1, G-protein coupled receptor 13, V28	microglia migration and adhesion	Jones et al. (2010)
*TMEM 119*	transmembrane protein 119	osteoblast induction factor (OBIF)	uncertain	Haynes et al. (2006) and Satoh et al. (2016)
*F4/80*	cell surface glycoprotein F4/80	adhesion G protein-coupled receptor E1, EGF-like module-containing mucin-like hormone receptor-like 1, EMR1	cell surface glycoprotein, expressed in mice, not confirmed in human	Lawson et al. (1990) Lin et al. (2005) and Roesch et al. (2018)
*FCER1G*	high-affinity immunoglobulin epsilon receptor subunit gamma	FcRgamma, Fc-epsilon RI-gamma, FceRI gamma	associates with pattern recognition, C-type lectin-like receptor [pattern recognition receptors (PRRs), C-type lectin-like receptor (CLEC)]; induces downstream signaling leading to maturation of APCs	Baker et al. (2014), Lorenz et al. (2015) and Mukherjee et al. (2019)
*FCRLS*	Fc receptor-like S, scavenger receptor		involved in microglial maintenance	Butovsky and Weiner (2018)
*Sirpα*	signal regulatory protein alpha	CD172a, SHPS-1, BIT	inhibitory receptor; interacts with a broadly expressed CD47; also called the “do not eat me” signal	Barclay and Van den Berg (2014) and Sierra et al. (2013)
*Siglec*	sialic acid-binding immunoglobulin type lectins		family of proteins, pro-inflammatory immune responses and phagocytosis are turned down in microglia by inhibitory Siglec signaling; less rodent homologs than human	Linnartz-Gerlach et al. (2014) and Smith and Dragunow (2014)
*Glut5*	glucose transporter 5	SLC2A5	exclusively microglial glucose transporter	Payne et al. (1997)
*P2Y12*	P2Y purinoceptor 12	ADPG-R, P2T(AC), P2Y(AC), P2Y(cyc), P2Y12 platelet ADP receptor, short name: P2Y(ADP), SP1999	detecting nucleotides like ATP released during injuries	Haynes et al. (2006) and Amadio et al. (2014)
**Intercellular proteins**				
*Pu.1*	transcription factor Pu.1	Spi- 1	plays a crucial role in determining macrophage lineages and microglial genesis and is a major factor in selecting the set of enhancers expressed by microglia.	Gosselin et al. (2014) and Butovsky and Weiner (2018)
*IBA-1*	ionized calcium binding adapter molecule 1	allograft inflammation factor 1 (AIF-1), microglia response factor (MRF-1), daintain	reorganization of microglial cytoskeleton, supporting the phagocytosis process	Sasaki et al. (2001)
*HexB*	β-hexosaminidase subunit β	N-acetyl-beta-glucosaminidase subunit beta	responsible for the degradation of GM2 gangliosides and other molecules containing terminal N-acetyl hexosamines	Butovsky et al. (2014)
*VIMENTIN*	vimentin	fibroblast intermediate filament	key controller for microglia activation	Jiang et al. (2012)
*Ferritin*	ferritin		responsible for iron storage and its homeostasis, which is downregulated in inflammation	Holland et al. (2018)
*Sall1*	Sal-like protein 1		maintenance of microglia homeostasis; its inactivation resulted in the conversion of microglia from resting tissue macrophages into inflammatory phagocytes	Buttgereit et al. (2016) and Butovsky and Weiner (2018)
**Activated state markers Membrane proteins**				
*CD16*	cluster of differentiation 16	low-affinity immunoglobulin gamma Fc region receptor III	Fc receptor detecting immunoglobulin gamma (IgG) antibodies; engaged in phagocytosis processes	Nagarajan et al. (1995) and Kigerl et al. (2009)
*CD32*	cluster of differentiation 32	low-affinity immunoglobulin gamma Fc region receptor II	membrane receptor for the Fc region of IgG; induces inflammatory signals	Kigerl et al. (2009)
*CD40*	cluster of differentiation 40	tumor necrosis factor receptor superfamily member 5	transduces signals activating ERK kinase	Lebedeva et al. (2005)
*CD86*	cluster of differentiation 86	T-lymphocyte activation antigen CD86 or B7–2	membrane co-stimulatory receptor responsible for immune cell proliferation and IL-2 production	Lebedeva et al. (2005)
*MHC II*	major histocompatibility complex II		mobilizes immune cells to inflammatory response in pathological situation; reacts to TGFβ1 in rodent but not in human	Lebedeva et al. (2005) and Smith and Dragunow (2014)
*CD163*	cluster of differentiation 163	scavenger receptor cysteine-rich type 1 protein M130, hemoglobin scavenger receptor	clears oxidative Hb, which in consequence leads to subsequent degradation of heme by heme oxygenase-1 (HO-1); produces Fe2+, CO and the anti-inflammatory metabolites	Etzerodt and Moestrup (2013)
*CD206*	cluster of differentiation 206	macrophage mannose receptor 1 (MRC-1), C-type lectin domain family 13 member D	endocytosis processes *via* detection of pathogenic glycoproteins and polysaccharide chains	Park et al. (2016) and Ohgidani et al. (2017)
**Intercellular proteins**				
*TSPO*	translocator protein	peripheral benzodiazepine receptor (PBR)	immunomodulation, regulation of apoptosis, cell proliferation	Pannell et al. (2020)
textitiNOS	inducible nitric oxide synthase	hepatocyte NOS, NOS type II, Peptidyl-cysteine S-nitrosylase NOS2	enzyme producing NO from L-arginine, promotes response against tumors and pathogens *via* NO production; promotes synthesis of inflammatory factors (IL-6) and is related with expression of transcription factors, e.g., IRF-1 and NF-κB	Vuolteenaho et al. (2009), Sierra et al. (2014) and Bogdan (2015)
*ARG1*	arginase 1	type I arginase, liver-type arginase	enzyme converting an amino acid arginine into ornithine and urea further metabolized to proline and polyamides; needed for wound healing or tissue remodeling, expressed in mice, not confirmed in human	Hesse et al. (2001), Munder (2009), Munder et al. (2005), Quirié et al. (2013), Franco and Fernández-Suárez (2015)
*Ym1*	chitinase-like protein 3	beta-N-acetylhexosaminidase Ym1, Chitinase-3-like protein 3, Chi3l3, ECF-L, Eosinophil chemotactic cytokine	heparin-binding lectin; prevents degradation of extracellular matrix components; expressed in mice, not confirmed in human	Pauleau et al. (2004), Odegaard et al. (2007), Odegaard et al. (2008) and Franco and Fernández-Suárez (2015)
*FIZZ1*	restin-like alpha	cysteine-rich secreted protein FIZZ1, RELMalpha	mediates interactions between sensory nerves and inflammatory cells in lung; blocks nerve growth factor-induced survival of dorsal root ganglion neurons; expressed in mice, not confirmed in human	Pauleau et al. (2004), Odegaard et al. (2007), Odegaard et al. (2008) and Franco and Fernández-Suárez (2015)
**Extracellular proteins**				
*MMP9*	matrix metalloproteinase 9	gelatinase B	regulates bioavailability of cytokines and chemokines in inflammation; promotes pro-inflammatory IL-1β maturation	Könnecke and Bechmann (2013) and Nissinen and Kähäri (2014)
*MMP12*	matrix metalloproteinase 12	macrophage metalloelastase	regulates bioavailability of cytokines and chemokines in inflammation	Nissinen and Kähäri (2014)

*As a reference for the protein name synonyms, www.uniprot.org was used*.

Many of the marker proteins described are expressed by both resting and activated microglial cells (see below). The recognition of those two phenotypes is, however, possible when comparing amounts of detectable protein levels (marked as “high” or “low”), as their expression changes due to activation. Semi-quantitative analysis of the expressions of two or even more markers and comparison of their ratios is now often used to discriminate particular types of microglial cells and is becoming good practice.

## Heterogeneity of Microglia

Although adult microglial cells are considered rather functionally homogenous, they show a broad spectrum of intrinsic heterogeneity (Stratoulias et al., 2019; Tan et al., 2020).

The first parameter of such heterogeneity regards the microglia ratio/density in the tissue, which varies in different regions, being ca. 5% in the cerebral cortex or cerebellum and ca. 12% in mouse substantia nigra (Lawson et al., 1990). The density of microglia has been reported in descending order in the forebrain, midbrain, and hindbrain, with cerebellum containing the fewest of them. Interestingly, a higher amount of microglial cells is reported in mouse gray matter than in the white matter (Lawson et al., 1990). The gradient pattern is parallel to the ontogenetic rodent CNS development.

The second parameter describing microglial heterogeneity is their cell morphology (Das Sarma et al., 2013). One of the earliest studies proposed division into: (i) compact cells with a small, round body and short, thick processes; (ii) longitudinally branched cells with an elongated body and long processes, often orientated parallel to the closest axons; and (iii) radially branched cells with a radial body and many branches (Lawson et al., 1990). In reality, microglial morphology shows tremendous variability and depends strongly on activation state and localization.

The third parameter of microglial heterogeneity is the regional difference in the set of protein markers expressed by cells both under homeostatic and pathologic conditions. For example, the expressions of markers involved in microglial activation, such as CD68, CD86, CD45, CX3CR1, CD11b, and HLA-DR, were reported to be higher in the human subventricular zone and thalamus (Böttcher et al., 2019). The microglial subpopulations of the temporal and frontal lobe, on the other hand, expressed lower CD206 levels while activated than other brain regions (Böttcher et al., 2019). Therefore, microglia can be stratified based on a specific set of diverse transcripts/proteins, often also corresponding with its localization in the brain (Stratoulias et al., 2019; Masuda et al., 2020; Tan et al., 2020).

### Differences Between Human and Rodent Microglial Protein Expression

The important issue in preclinical studies of immunological response is the difference between the microglial markers expressed in rodents and human (Martinez and Gordon, 2014). This often makes it difficult to translate basic study results to human disease (Jubb et al., 2016). Whole-genome studies indicated greater microglia diversity in the human brain when compared to mouse microglia (Gosselin et al., 2014; Zhong et al., 2018; Masuda et al., 2019; Sankowski et al., 2019).

The main differences between human and rodent microglia arise from: (a) the rodent pathogen-free laboratory conditions vs. human lifetime exposure to multiple pathogens; (b) life span further affecting the number of immune system challenges; (c) similarity of experimental rodent genome due to breeding vs. extreme genomic variability in humans; (d) differences in anatomy: the majority of rodent microglia are localized in gray matter vs. the majority are in white matter in humans; (e) inflow of peripheral macrophages to the CNS is confirmed in rodents, but circumstances in human are not so certain (Boche et al., 2013). These aspects cause higher spatial and temporal diversity of microglia in human brain when compared to microglia from laboratory mice (Böttcher et al., 2019), especially in the aging or diseased brain (Smith and Dragunow, 2014; Galatro et al., 2017).

Caution should be taken when using human vs. rodent microglia expressed molecules (Smith and Dragunow, 2014; Franco and Fernández-Suárez, 2015; Roesch et al., 2018). For example, TLR4 expression is high in rodent but low in human. IFNγ receptor is not detected on microglia in human tissue. The Siglec (Sialic acid-binding immunoglobulin-type lectin) family is smaller in rodent than in human; especially, Siglec-11 has no homolog in rodent, and Siglec-3 (CD33) shows substantial species difference. Also, MHCII reacts to TGFβ1 in rodent but not in human. F4/80, Ym1, FIZZ1, and arginase 1 (ARG1) were not confirmed in human tissue.

### Other Aspects of Variability to Consider

Other variables in the literature suggest functional differences between male and female microglia in a variety of disease contexts (Hammond et al., 2019). Another newly described variable between microglial subsets worth mentioning is the rate of microglial self-renewal (Réu et al., 2017; Tay et al., 2017). The differences between developmental and adult brain microglia, as well as aging, add substantially to the microglial heterogeneity but are out of the scope of this review.

## How to Discriminate Innate from Infiltrated Macrophages

Resident CNS microglia and macrophages from the periphery have different origins; however, the precise distinction between them in tissue is difficult because they share a majority of markers such as CD11b, F4/80, CX3CR1, CD45, and IBA-1 (Amici et al., 2017; Figure 1).

One of the distinct molecules is the CD44 marker reported to be expressed only by infiltrating cells and not on resident microglia (Bennett et al., 2016). Another study examining the gene transcription of adult microglia compared to peripheral cells has suggested that microglia lack CD169 (Butovsky et al., 2012). Siglec-H was also indicated as a marker for microglia in mice, absent from CNS-associated macrophages and CNS-infiltrating monocytes except for a minor subset of cells (Konishi et al., 2017).

### Quantitative Markers

In some cases, the quantitative differentiation of markers is advised. For example, in the adult brain, subtle differences in CD45 protein amount can be detected between microglia expressing CD11b^+^/CD45^low^ and macrophages expressing CD11b^+^/CD45^high^ (Ford et al., 1995; Zhang et al., 2002; Grabert et al., 2016), although caution must be taken because CD45 expression increases in microglia upon inflammation and with aging (Roesch et al., 2018; Haage et al., 2019; Benmamar-Badel et al., 2020).

A comparison of different phenotypes of human CNS-resident microglia and peripheral immune cells showed characteristic patterns of markers. Three main markers were chosen to distinguish perivascular macrophages (CD11b^+^/CD206^high^/CD163^+^) from resident microglia (CD11b^+^/CD206^low/−^/CD163^−^). It has to be noted that, despite undetectable levels in physiological conditions, CD206 and CD163 are expressed in the activated M2 anti-inflammatory microglial phenotype (Böttcher et al., 2019; see below).

### Ontogeny and Transcription Factors

Transforming growth factor β (TGFβ)-dependent signaling was proposed to allow microglia to be distinguished from peripheral macrophages and other immune cells in animal models. This includes expression of several factors, such as FCRLS (Fc receptor-like S, scavenger receptor), HexB (β-hexosaminidase subunit β), P2Y12R, or TMEM119 (Butovsky et al., 2014; Gosselin et al., 2014; Lavin et al., 2014; Bennett et al., 2016; Buttgereit et al., 2016; Satoh et al., 2016; Butovsky and Weiner, 2018). It was shown that the development of microglia and maintenance of their identity relies on the transcription factors SALL1 and Pu.1 (Buttgereit et al., 2016; for an in depth review see Yeh and Ikezu, 2019). This was confirmed in human microglial cells, which exclusively expressed P2Y12R and TMEM119 with additional high expression of CD64, CX3CR1, TGFβ, TREM2, CD115, CCR5, CD32, CD172a, and CD91 and low to absent expression of CD44, CCR2, CD45, CD206, CD163, and CD274 (PD-L1; Böttcher et al., 2019). The transcription factors Pu.1 and Myb were also indicated to allow microglia (Pu.1-dependent transcription) to be distinguished from peripheral macrophages (Myb-dependent; Schulz et al., 2012; Gosselin et al., 2014; Lavin et al., 2014; Bennett et al., 2016; Butovsky and Weiner, 2018). P2Y12R is a metabotropic purinoceptor detecting nucleotides like ATP, being released during injuries. It is considered along with TMEM119 as one of the most specific microglial markers, expressed only by yolk sac-derived cells (Haynes et al., 2006; Amadio et al., 2014).

The additional significant difference between microglia and macrophages that helps in their discrimination during the CNS inflammation is that microglia activation is detectable very quickly (within 24 h) while peripheral macrophage infiltration is detectable within the next few days (Schilling et al., 2003).

## Microglia Activation

Microglial cells have different states depending on the actual tissue needs, and two main states can be underlined: resting and activated.

In normal, healthy conditions, microglia are quiescent. They are also called resting (Figure 1), but the truth is that the cells are very motile and are constantly surveilling the local environment with their processes. Due to their shape, they are sometimes called ramified microglia. They have small, round cell bodies with little cytoplasm and intensive branching processes (Davalos et al., 2005; Nimmerjahn et al., 2005). This is the dominant state if there are no pathological signals in the surrounding environment (Lawson et al., 1992; Banati, 2003).

**Figure 2:**
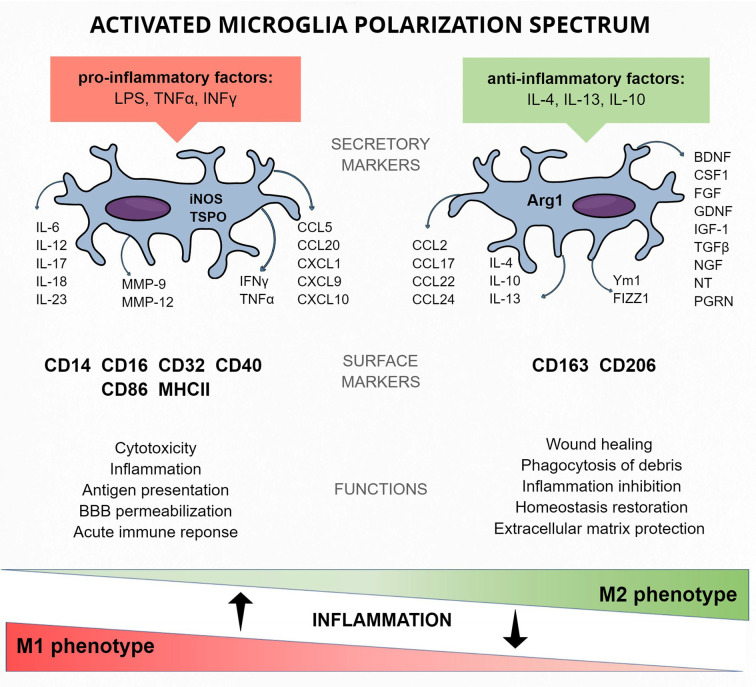
Activated microglia protein markers of the M1 and M2 polarization spectrum—cellular and released.

If quiescent microglial cells spot any potentially dangerous signals or molecules or a lack of normal signaling coming from neurons and other glial cells, they undergo morphological and functional change into amoeboid, activated microglia (Das Sarma et al., 2013; Figure 2). Phenotypically, those consist of a round cell body with short, thick pseudopodia that enable them to move quickly towards the danger zone, release cytotoxic substances to kill the pathogen, and perform phagocytosis (Dihne et al., 2001). Resting and activated are the two opposite morphological types that border the wide spectrum of in-between phenotypes depending on the strength of activation and time-line of inflammation process.

### Markers Involved in Mechanisms of Microglia Activation

Microglia may be activated by various factors present in their surroundings and spotted during the “surveilling” process. Those factors may be exogenous, such as pathogen-associated molecular patterns (PAMPs), bacterial LPS, or pathogen genetic material or viruses. Activating signals can also be endogenous, presented by stressed surrounding cells such as danger/damage-associated molecular patterns (DAMPs, for example, nucleotides) and protein aggregates like amyloid β (Aβ) senile plaques (Kreutzberg, 1996; Stence et al., 2001) or can be released by other microglial cells and astrocytes. Recognition of harmful vs. healthy signals is the most complex step, as it requires microglia to distinguish very specific information and not fight the healthy tissue, as happens in some CNS diseases. This requires “find-me” signals released by pathogen or apoptotic cells and specific receptors present on microglia [e.g., vitronectin receptor (VNR), MER receptor tyrosine kinase (MerTK), CX3CR1, or complement receptor 3 (CR3)]. Microglia also respond to so-called “eat-me” signals from the target cells. These are phosphatidylserine or calreticulin present in, e.g., disrupted neuronal membrane, secreted fractalkine or opsonins (growth arrest-specific 6, Gas6), milk fat globule epidermal growth factor 8 (MFG-E8), or complement factors. In opposition, the target cells can also send “do not eat me” signals protecting them from being phagocytosed by microglia. Those signals are recognized by specific microglial receptors. For instance, neuronal CD47 and sialylation are cell-surface proteins recognized by microglial signal regulatory protein α (SIRPα; Barclay and Van den Berg, 2014) and Siglec receptors, respectively (Linnartz-Gerlach et al., 2014).

### Microglia Cytotoxicity

The cytotoxic factors released by activated microglia are peroxynitrite, hydroxyl radical or hypochlorous acid, the NO and superoxide metabolites (Ghosh et al., 2018). The final stages of phagocytosis are engulfment and digestion, leading to the internalization of the target and its complete degradation in mature phagolysosome (for reviews of the mechanism see Sierra et al., 2013; Vilalta and Brown, 2018). As well as for infections, phagocytosis is also necessary in case of tissue injury, when there is a need to remove debris and damaged cells. In the context of this review, it is worth mentioning that extended and prolonged release of cytotoxic substances may also influence healthy cells, inducing inflammation and prompting astrocytes to become neuro-aggressive (Liddelow et al., 2017).

### Microglia Proliferation

The activation of microglia is directly connected with their proliferation, enhanced production, and expression of inflammatory factors and surface proteins (Graeber, 2010). Recent studies confirmed that activated microglia have enhanced expression of proliferation markers from different cell-cycle phases: Ki-67 (G1, S and G2 phase, mitosis), cyclin A (S and G2 phase, mitosis), and cyclin B (mitosis; Böttcher et al., 2019). Similarly to the peripheral macrophages, microglia also present antigens (fragments of phagocytosed, digested pathogens) using major histocompatibility complex II (MHC II) molecules. This induces an inflammatory response in the closest microglial cells. In the alert situation, distant microglia also support the local population by infiltrating from other brain regions. Therefore, during inflammation, the number of microglial cells increases rapidly in the infected region to fight the danger as rapidly as possible.

### The Spectrum of Microglia Activation Phenotypes and Their Functions

Various stimuli are responsible for sundry activations of microglia. This allows the distinction of the classical M1 phenotype, activated mainly by pathogens and pro-inflammatory factors [e.g., LPS, tumor necrosis factor alpha (TNFα), interferon gamma (IFNγ)] from the alternative M2 phenotype, activated by anti-inflammatory factors (e.g., IL-4, IL-10; Jang et al., 2013; Figure 2).

The names of the M1/M2 phenotypes came by analogy with the first peripheral immune system cells, the polarization phenotypes of which were called T helper (Th) cells (classical Th1 and alternative Th2 phenotype; Biswas and Mantovani, 2010; Martinez and Gordon, 2014). By analogy, peripheral monocyte-macrophage lineage cells were proven to undergo polarization into M1 and M2 phenotypes under the same conditions (Mantovani et al., 2002). Classical M1 blood cell-derived macrophage polarization is activated by the TLR- and IFN-mediated signal transducer and activator of transcription 1 (STAT1) signaling pathway. In consequence, transcription factor interferon regulatory factor 5 (IRF5) undergoes upregulation and stimulates the production of pro-inflammatory factors such as IL-12, IL-23, and TNF (Krausgruber et al., 2011). Alternative M2 macrophages, on the other hand, engage the STAT6 pathway *via* IL-4 and IL-13 induction or STAT3 *via* IL-10 (Lang et al., 2002). This pathway induces CD206 (also known as mannose receptor MRC1), FIZZ1 (also known as resistin-like α), or Ym1 (known as chitinase 3–like 3, CHI3L3), peroxisome proliferator-activated receptors gamma and delta (PPARγ, PPARδ), and ARG1 (Pauleau et al., 2004; Odegaard et al., 2007, 2008). When microglia gained interest in the context of pathological inflammation, the same phenotypes and activation pathways were proposed (Mills et al., 2000; Murray et al., 2014).

M1 activation of microglia is considered as aggressive, leading to cytotoxicity and robust, immediate inflammation related to the release of pro-inflammatory cytokines and chemokines (e.g., TNFα, IL-6, IL-1β). Activated microglia express NADPH oxidase [generating superoxide and reactive oxygen species (ROS)], iNOS, MMP-12 (matrix metalloproteinase 12), MHCII, Fc receptors, and integrins. Microglia can be activated by Th cells releasing IFNγ or by bacterial LPS (Murphy et al., 2010; Lively and Schlichter, 2018).

Switching the activation phenotype to M2 can be assumed to have a silencing effect, leading to the reintroduction of environmental homeostasis and promoting recovery (Murray et al., 2014). M2 activation is induced by the presence of IL-4, IL-13, or IL-10, ligation of Fc receptors, or activation of PPARγ transcription factor (Saijo et al., 2013). The effect of this activation is release of anti-inflammatory cytokines (such as IL-10 and TGFβ), growth factors (insulin-like growth factor I, fibroblast growth factor), colony-stimulating factor 1 (CSF-1), neurotrophic factors (nerve growth factor, brain-derived neurotrophic factor, neurotrophins 4/5, glial cell–derived neurotrophic factor) and pro-survival factor progranulin.

M1 and M2 phenotypes can be assumed as opposite phenotypes in a broad activation spectrum. In tissue, many intermediate states exist (for a review see Liddelow and Barres, 2017). Such strict classification may refer to *in vitro* conditions with selected stimulation but have no representation in the *in vivo* tissue environment (Martinez and Gordon, 2014).

The process of inflammation is strictly regulated, starting from the robust activation but also concluding in its resolution and tissue repair. Therefore, the subtypes of microglial phenotype seamlessly pass from one to another, actually making a gradient of phenotypes in the tissue.

## Common Markers of Activated Microglia

Microglial cells are the first response in active protection in situations related to homeostasis disruption. These cells are equipped with a large number of tools necessary for both the recognition and destruction of threats. These consist of TLRs, nucleotide-binding oligomerization domain-like receptors (also named NLRs), scavenger receptors, MHC complexes, intracellular signaling pathway activation, inflammatory and cytotoxic factor production and release, and phagocytosis (Boche et al., 2013). All of these can be used as molecular markers of activated microglia.

### Antigen Presentation-Related Markers

Microglia are antigen-presenting cells, so while activated, they use MHC II molecules to present fragments of phagocytosed, digested pathogens, which triggers further inflammatory response in surrounding microglial cells and spreads the inflammation. Also, other membrane molecules necessary for antigen presentation undergo upregulation on the cell surface. CD40 (tumor necrosis factor receptor superfamily member 5) induces signal-regulated kinase (ERK) activation and immune factor secretion (Lebedeva et al., 2005). MHC II binds the peptide, but the full activation of the immune cell also requires signals from co-stimulatory receptors. For instance, CD80 and CD86 (also known as B7–1 and B7–2, respectively), CD28, and intercellular adhesion molecule 1 (ICAM1) generate co-stimulatory signals after MHCII activation (Park et al., 2003; Lebedeva et al., 2005).

### Transmembrane and Surface Proteins

The transmembrane and surface proteins are the first line of pathogen recognition. The popular microglia marker protein CD11b (also known as integrin alpha M, ITGAM; complement receptor 3 alpha, CR3A) is an alpha subunit that, along with CD18 (also named integrin beta chain-2, ITGB2), constructs an integrin complement receptor 3 (CR3, also known as macrophage 1 antigen, MAC-1). It is involved in adhesion processes and uptake of complement-coated molecules. This protein is also present on the membranes of leukocytes, so it is not a specific marker of resident microglia. It is commonly used in preclinical studies but, in the majority, with the use of antibody against OX-42 (name of the clone reacting with CD11b epitope; Chakrabarty et al., 2010; Jeong et al., 2013). Other transmembrane and surface proteins of activated microglia are CD68, CD16, CD14, CD45, CA115, CX3CR1, F4/80, and FCER1G. CD68 receptor, macrosialin, is a transmembrane protein localized in cellular, lysosomal, and endosomal membranes of monocytes and macrophages/microglia. This protein level is strongly upregulated during inflammation and has the ability to internalize from the cell surface to endosomes immediately after stimulation (Holness and Simmons, 1993; Kurushima et al., 2000; Fadini et al., 2013). CD16 (low-affinity immunoglobulin gamma Fc region receptor III-B) is an Fc receptor detecting IgG antibodies and engaged in phagocytosis processes (Nagarajan et al., 1995). CD14 is a co-receptor for transmembrane TLR4 and endosomal TLR7/9, presenting antigens to them (Baumann et al., 2010; Zanoni et al., 2011). The structure of CD45 (also known as receptor-type tyrosine-protein phosphatase C, PTPRC) includes an enzymatic subunit, and CD45 is a positive regulator of T-cell activation (Rice et al., 2017). CD115 (macrophage colony-stimulating factor 1 receptor, M-CSF-R, or colony-stimulating factor 1 receptor, CSF-1R) belongs to the cell-surface receptor tyrosine kinase family. CD115 recognizes pro-inflammatory ligands like IL-34 or CSF-1, which are cytokines controlling the proliferation, differentiation, and general functioning of macrophages/microglia. This receptor is involved in innate immunity response and in the reorganization of actin cytoskeleton, which contributes to the phenotype change and infiltration of inflamed regions (Jenkins et al., 2013). Importantly, it has been proven that CD115 depletion or inhibition leads to robust microglia death (Elmore et al., 2015, 2018). Protein CX3CR1 is a transmembrane, G-coupled CX3CL1 (fractalkine) receptor mediating its functions related to microglia migration and adhesion (Jones et al., 2010). The cell surface glycoprotein, F4/80 (also known as adhesion G protein-coupled receptor E1 or EGF-like module-containing mucin-like hormone receptor-like 1, EMR1), is described as one of the most specific markers of murine macrophages and microglia (Lawson et al., 1990; Lin et al., 2005) while its expression in human was not confirmed. FCER1G is high-affinity IgE receptor, which associates with pattern recognition, C-type lectin-like receptor CLEC4D, and CLEC4E. This induces downstream signaling leading to the maturation of antigen-presenting cells. This molecule is also probably involved in aging and neurodegenerative processes (Baker et al., 2014; Lorenz et al., 2015; Mukherjee et al., 2019).

### Intracellular and Effector Proteins

Activation of surface receptors and other proteins triggers the mobilization of intracellular signaling pathways and effector proteins, which can also serve as markers of microglia activation and are often upregulated in the process. iNOS is an enzyme producing NO from L-arginine. Its functions in organisms are diverse, but in microglia, it promotes response against tumors and pathogens *via* NO production. iNOS promotes the synthesis of inflammatory factors (IL-6) and is related with expression of transcription factors (e.g., IRF-1 and NF-κB), all of which are known to be involved in the microglia inflammatory response (Vuolteenaho et al., 2009; Sierra et al., 2014; Bogdan, 2015). The level of iNOS in quiescent glia is almost undetectable.

The most commonly used protein marker of microglia activation is an elevated level of IBA-1. This is a member of the calcium-binding protein group. It can be found under other names also: allograft inflammation factor 1 (AIF-1), microglia response factor (MRF-1), or daintain. IBA-1 is an intracellular protein, and its functions are related to the reorganization of microglial cytoskeleton and support of the phagocytosis process. The latter is possible thanks to its ability to bind actin molecules (Sasaki et al., 2001). This protein is one of the most widely examined in biochemical studies because of its conservative amino acid sequence and stability of antigenic epitopes through different species, including human (Yun et al., 2018). Vimentin, the major intermediate filament, is also used as a general marker of microglia (or macrophages). During inflammation, it is cleaved by calpains into short fragments that shuttle signaling molecules (like MAP-kinases) to the nucleus (Perlson et al., 2005). In microglia, vimentin was reported to be necessary for cellular activation, and it seems to play an essential role in preventing neuronal damage in animal models (Jiang et al., 2012). Its role in inflammation was also described in astrocytes (Pekny and Nilsson, 2005), so it cannot be used as a specific microglial marker. Another important microglial multimeric complex protein is ferritin, which is responsible for iron storage and its homeostasis and is upregulated due to microglia activation (Holland et al., 2018). Interestingly, microglial iron transport and homeostasis pathways are differentially active in response to pro- and anti-inflammatory stimuli. For a review, see Nnah and Wessling-Resnick (2018).

### Secreted Molecules

Equally important as the surface proteins are those secreted by reactive microglia. Cytokines, including interleukins (e.g., IL-1β, IL-6), TNFα, IFNγ, chemokines (CCL2, CX3CL1, CXCL10), glutamate, and NO act as transmitters in inflammation. NO is also a toxin against pathogens. Among enzymes, cathepsins are released proteases supporting inflammation driven by microglia (Lowry and Klegeris, 2018), and matrix metalloproteinases regulate the bioavailability of cytokines and chemokines in inflammation (Nissinen and Kähäri, 2014). Some of them, like MMP-9, can be used as active microglia markers.

## M1/M2 Phenotype Markers

### M1 Phenotype-Related Markers

The relation between Th1/M1 and Th2/M2 cells is also reflected in the similar factors released by them (Mills et al., 2000; Figure 2). It was reported that IFNγ produced in Th1 cells induces M1 microglia polarization and proliferation, and also, Th2 cells were reported to activate M2 polarization of microglia by secreting anti-inflammatory IL-4 (Edwards et al., 2006). This, however, unlike in M1, does not lead to microglia proliferation (Jenkins et al., 2013).

The classical M1 microglia response to pathological states is connected with pro-inflammatory factor production and release. iNOS metabolic enzyme contributes to NO synthesis, and its levels become strongly elevated during inflammation (Quirino et al., 2013). Its role was also underlined in the general activation markers section. M1 polarization phenotype can be recognized by the detection of surface receptors. CD16 and CD32 are membrane receptors for the Fc region of IgG, and their role is to induce inflammatory signals (Kigerl et al., 2009). Levels of CD86 (also known as T-lymphocyte activation antigen CD86 or B7–2), a membrane co-stimulatory receptor responsible for immune cell proliferation and IL-2 production, as well as CD40, are upregulated in activated M1 microglia. They were both also mentioned as general and active microglia markers, but their increased expression level can help discriminate the phenotype. MHC II complex mobilizes immune cells to inflammatory response in pathological conditions. Activation of the membrane proteins described above leads to enhanced production and secretion of immune factors, which also can be treated as M1 phenotype markers. Pro-inflammatory cytokines (such as the IL-1 family, IL-6, IL-12, IL-17, IL-18, IL-23, TNFα, and IFNγ) are responsible for the maintenance of inflammation (Biswas and Mantovani, 2010; Kalkman and Feuerbach, 2016). The role of chemokines (e.g., CCL5, CCL20, CXCL1, CXCL9, CXCL10) is to recruit immune cells. MMP-9 promotes pro-inflammatory IL-1β maturation (Könnecke and Bechmann, 2013).

### M2 Phenotype-Related Markers

The role of the alternative M2 active microglia phenotype is to stop the inflammation and restore homeostasis to the surroundings (Varin and Gordon, 2009). In contrast to the classical activation path, M2 microglia release anti-inflammatory factors and produce proteins protecting extracellular matrix, contributing to wound healing or phagocytosis of debris (Martinez et al., 2009). The surface M2 specific protein markers include CD206, a receptor localized in cellular and endosomal membranes that is responsible for endocytosis processes *via* detection of pathogenic glycoproteins and polysaccharide chains (Park et al., 2016; Ohgidani et al., 2017). The hemoglobin scavenger receptor CD163 is responsible for clearing oxidative hemoglobin, which, in consequence, leads to subsequent degradation of heme by heme oxygenase-1 (HO-1) and production of Fe^2+^, CO, and the anti-inflammatory metabolites (Etzerodt and Moestrup, 2013). The anti-inflammatory cytokines are also used as M2 phenotype markers: IL-1 receptor antagonist (IL-1Ra), IL-4, TGFβ, and IL-10, as well as common IL-4 and IL-13 receptor antagonist (IL-4Ra). Similarly, chemokines (e.g., CCL2, CCL22, CCL17, CCL24) are secreted by M2 microglia in order to shut down the ongoing inflammation (Biswas and Mantovani, 2010). Secretory proteins Ym1 and FIZZ1 are factors whose release is dependent on the levels of anti-inflammatory cytokines IL-4 and IL-13 (Raes et al., 2002; Du et al., 2017). ARG1 is an enzyme converting an amino acid arginine into ornithine and urea, further metabolized to proline and polyamides needed for wound healing or tissue remodeling (Hesse et al., 2001; Munder et al., 2005; Munder, 2009; Quirié et al., 2013). It is good practice to compare the ratio of the above-mentioned M1 marker iNOS and ARG1 in activated microglia because these two factors compete for the same substrate—arginine. The overexpression of ARG1 leads to downregulation of NO production and iNOS expression, which enables M1/M2 distinction (Corraliza et al., 1995).

To identify the predominance of M1 or M2 phenotype in a tissue or culture, the ratios of marker amounts of both states can be compared, e.g., IL-12 released by M1 microglia and IL-10 released by M2 cells. In such a comparison, the ratio IL-12^high^/IL-10^low^ could be a confirmation of M1 activation (Mantovani et al., 2004). The CD14/CD16 expression ratio can also be compared to distinguish the classic, pro-inflammatory M1 activation pattern (CD14^high^/CD16^−^) from alternative anti-inflammatory M2 (CD14^low^/CD16^+^; Fadini et al., 2013).

The additional division into transitional microglia phenotypes (called in the literature M2a, M2b, M2c, $\text{M}1\frac{1}{2}$, or “intermediate” microglia) was characterized as expressing markers for both M1 and M2 at the same time, like CD86^+^/CD206^+^, or M1 macrophages expressing specific markers like MHCII and CD86 and lacking M2 markers FIZZ1 and Ym1 but expressing the typical IL-10^high^/IL-12^low^ M2 cytokine profile (Edwards et al., 2006; Filardy et al., 2010; Murray et al., 2014; Knudsen and Lee, 2016; Zhou et al., 2017). Up to the present, studies regarding intermediate microglia polarization phases are not unified enough to demarcate clear borders between them (Mills et al., 2000; Martinez and Gordon, 2014; Murray et al., 2014).

## Energy Metabolism-Related Microglia Markers

Another class of microglia markers can be found among the energy metabolism proteins. For example, the glucose transporter GLUT5 (SLC2A5), which is classified, however, as a fructose transporter because of its affinity levels, is exclusively microglial (Payne et al., 1997).

Quiescent microglia rely primarily on oxidative phosphorylation for ATP production (Moss and Bates, 2001; Chénais et al., 2002; Orihuela et al., 2016). Shifting the phenotype from quiescent to activated also requires a fast adaptive energy metabolism change. This could be used as an additional, non-specific, quantitative marker of microglia phenotype. Studies of peripheral immune cells have long ago demonstrated that polarization to an M1 phenotype is often accompanied by a shift in cell energy production to aerobic glycolysis, while M2 correlates with the use of mitochondrial oxidation (Orihuela et al., 2016; Fumagalli et al., 2018). The literature still lacks exact *in vivo* studies from resident brain microglia, but *in vitro* data confirm this observation (Voloboueva et al., 2013; Gimeno-Bayón et al., 2014). What we know, however, is that pro-inflammatory activated M1 macrophages increase their glucose uptake and lactate production with activation of the pentose phosphate pathway and decreased mitochondrial oxygen consumption, allowing for fast oxidative bursts of NO and superoxide to kill targets (Orihuela et al., 2016). Continuous metabolism of glucose by the hexose monophosphate shunt is required for the supply of NADPH substrate (Cohen and Chovaniec, 1978; Decoursey and Ligeti, 2005).

On the contrary, in the anti-inflammatory M2 macrophages, glucose consumption is significantly lower than in M1, and they can also utilize fatty acid oxidation, contributing to phagocytosis by regulating membrane fluidity (Orihuela et al., 2016; Amici et al., 2017). Therefore, glycolysis vs. oxidative phosphorylation marker ratios combined with microglia-specific proteins can also be used as non-specific markers of microglia phenotype.

One of the popular mitochondrial markers is translocator protein (TSPO), which was first described as peripheral benzodiazepine receptor. It is localized on the outer mitochondrial membrane of the majority of cell types. Activation of rat microglia *in vitro* by LPS and IFNγ increases the amount of mitochondria (Banati et al., 2004; Ferger et al., 2010) and is associated with increased expression of TSPO (Venneti et al., 2006). It is used now in clinical brain imaging techniques as a marker of activated glia because of its strongly enhanced expression during neuroinflammation. Recent studies confirmed that TSPO can serve as an excellent pro-inflammatory activation marker (Pannell et al., 2020).

## Examples of Microglial Markers in Aging and Human Brain Diseases

### Aging

Aging is an important factor influencing microglia functioning and expressed markers, often contributing to neurodegenerative disease pathology and enhancing the risk of Parkinson’s disease, Alzheimer’s disease, dementias, synucleinopaties, etc. It may be interestingly defined as a situation where microglia lose their natural properties and become hyperactive and resistant to regulation. Morphologically, the ramification of aged microglia and their motility decrease, probably causing less efficient surveillance (Rozovsky et al., 1998). In addition, *in vitro* data indicate that aged microglia become less sensitive to anti-inflammatory regulatory signals, such as TGFβ or granulocyte-macrophage colony-stimulating factor (GM-CSF; Rozovsky et al., 1998). During their life span, episodes of systemic inflammation and cytokine stimulation can permanently increase their reactivity; this is called priming. At the same time, aging can lead microglia to gradually acquire a hypersensitive phenotype (Godbout et al., 2005). They express more MHC II molecules and have enhanced sensitivity to stimuli (Frank et al., 2006). All of these features are connected with the expression and secretion of characteristic inflammation mediators like CD68 or IL-1β (Frank et al., 2006; Choi et al., 2007; Schuitemaker et al., 2012; Norden et al., 2015). Interestingly, experimental repopulation of rodent brain microglia by temporal blocking of CSF-1R (known as CD115) activity resulted in the restoration of physiological, surveilling phenotype. Factors overexpressed by ageing microglia were reduced by repopulation. Unfortunately, the aged brain environment still forced the pro-inflammatory phenotype of “new” microglia (O’Neil et al., 2018). In parallel, microglia decreased their responsiveness to anti-inflammatory stimuli (Kumar et al., 2013). This may be related to decreased levels of IL-4Rα, scavenger receptor A, and the Aβ degradation enzymes (neprilysin, insulin-degrading enzyme, and MMP-9; Hickman et al., 2008; Fenn et al., 2012).

### Neurodegenerative Diseases

For physiological maintenance of homeostasis, the balance between rapid inflammatory response and its silencing has to be kept. In the case of microglia, M1 activation and pro-inflammatory factor release have to be counteracted by M2 activation. Otherwise, the consequences of a persistent cellular offensive are destructive to the surrounding neurons and other cells due to the prolonged M1 activation and cytokine and ROS release (Banati, 2003; Kigerl et al., 2009). Inflammation is being reported in the majority of diseases involving ongoing neurodegeneration like Parkinson’s disease and Alzheimer’s disease, amyotrophic lateral sclerosis, multiple sclerosis, neurotropic viral infections, stroke, paraneoplastic disorders, and traumatic brain injury (Lehnardt et al., 2003; Marshall et al., 2013; Walker and Lue, 2015; Mathys et al., 2017; Lowry and Klegeris, 2018), but precise mechanisms remain to be elucidated (Amor et al., 2010; Chitnis and Weiner, 2017).

Parkinson’s disease is related with progressive degeneration of nigrostriatal pathway dopaminergic neurons. Studies of Parkinson’s disease suggest that there is robust microglia activation with parallel inflammatory factor upregulation in the brain regions affected (Langston et al., 1999; Walker and Lue, 2015). Elevations of IL-1β, TNFα, ROS, and NO levels have been detected in the substantia nigra and corpus striatum, as well as in cerebrospinal fluid and serum (Mogi et al., 1994; Le et al., 2001). Interestingly, it was reported that IgG isolated from the sera of Parkinson’s disease patients have the ability to affect healthy dopaminergic cells in the substantia nigra, causing the death of 40% of them in mice (He et al., 2002). Engagement of NLRP3 inflammasome complex was reported in neuronal degeneration (Mohamed et al., 2015). Interestingly, this complex, which is responsible for caspase 1-dependent release of pro-inflammatory cytokines and cell death, may be necessary for M1 activation of microglia, indicating the role of this phenotype in Parkinson’s disease development (Gaikwad et al., 2017). Whether microglia activation or neuronal degeneration occurs first remains unknown, but an increasing volume of experimental results strengthen the first theory, including those that showed that non-steroid anti-inflammatory drugs lower Parkinson’s disease risk (Chen et al., 2003).

The morphological picture of Alzheimer’s disease shows robust microglia activation in parallel with Aβ senile plaque generation. Consequently, elevated levels of pro-inflammatory cytokines (IL-1β, IL-6, and TNFα), TMEM119, and iNOS were reported in patients (Haas et al., 2002; Satoh et al., 2016). Aβ plaques have been shown almost exclusively to induce an M1 response in Alzheimer’s disease animal models, with an additional high proliferation rate in response to the neurodegenerative state (Liu et al., 2012; Mathys et al., 2017). Researchers have divided reactive cells into the early and late response microglia and examined the differences in transcription patterns. Interestingly, the majority of the markers of those cell activations differed from the peripheral system macrophage markers, indicating that resident microglia were predominantly responsible for the observed differences (Liddelow et al., 2017; Mathys et al., 2017).

The characteristic feature of amyotrophic lateral sclerosis is a loss of motor neurons in adult life. According to current knowledge, microglial cells remain in the surveilling state during the early stages of amyotrophic lateral sclerosis development, and the activation of those immune cells observed later on in disease progression probably involves both M1 and M2 phenotypes coincidentally (Boillée et al., 2006; Liao et al., 2012; Chiu et al., 2013; Geloso et al., 2017; Volonté et al., 2019). This introduces a large spectrum of possibilities for putative pharmacotherapies, which may be targeted to silencing of neurodegenerative M1 activation or enhancing neuroprotective M2 activation.

As in Alzheimer’s disease, in multiple sclerosis, oxidation may induce demyelination of neuronal axons, and activated microglial cells are the major source of ROS burst (Gray et al., 2008; Fischer et al., 2012; Miller and Wachowicz, 2013). Interestingly, IBA-1^+^/CD68^+^ infiltrating macrophages did not express TMEM119 in demyelinating lesions of MS (Satoh et al., 2016). Similarly, single-cell analysis showed that TMEM119 was also downregulated or even absent, while expression of apolipoprotein E and MAFB increased (Masuda et al., 2019).

Microglia activation polarization apparently also plays a role in bipolar disorder. The M1/M2 ratio differs between manic and depressive states. M2 microglia were significantly downregulated in patients in the manic phase, based on CD206 expression (Ohgidani et al., 2017). Enhanced activation of the general microglial population was confirmed in patients using the PET method.

An interesting mechanism influencing microglia polarization was reported in a cancer study where patient glioma cells secreted anti-inflammatory factors (IL-10, IL-4, TGF-β, and PE2). In consequence, microglia activation was shifted into alternative, immunosuppressive M2, allowing cancer cells to avoid M1 microglia attack (Kikuchi and Neuwelt, 1983; de Martin et al., 1987; Frei et al., 2015; Nduom et al., 2015).

Although the determination of M1 or M2 phenotype is still challenging, TSPO expression in the microglia of patients seems to be a promising target to do so (Haarman et al., 2016; Pannell et al., 2020). PET imaging allows it to be determined, *in vivo* in patients, whether microglia undergo M1 or M2 activation in their condition. This is a great, promising opportunity to broaden basic studies into actual clinical conditions and to confirm whether changes observed on the biochemical level in laboratory models reflect the disease. Such information could accelerate the construction of therapies and their transfer to clinic.

## Summary

In this review, we summarized the main functions of microglia and their related markers. There are several core proteins that can be used as general microglia markers, whatever the microglial state. The broad aspects of microglial heterogeneity and the means of its recognition were presented to give an overview of the complexity of the subject. Caution should be taken while choosing markers appropriate for experimental species because of the differences between rodent and human markers. A few methods that allow selective discrimination of resident microglia from the peripheral macrophages are available at the moment, as well as multiple indicators of their activation and particular phenotypes. It is important to remember that M1/M2 indicators are the borders of a broad spectrum of intermediate phenotypes. Both qualitative markers and semi-quantitative estimates are useful, and we strongly recommend using several markers to precisely describe the cell type or state. The examples of markers used in the studies of inflammatory processes in the aging brain or in neurodegenerative diseases show tendencies to look for disease-specific microglial phenotypes and perspectives for broader use of marker molecules in future diagnostics.

The current experimental approach is directed towards single-cell transcriptomics and proteomic studies describing microglia-enriched markers and discovering new subtypes of microglia in a specified species, age, brain structure, and disease, hopefully allowing the creation of a cell-specific profile database that could be used to choose the best markers for each study. There are a plethora of proteins that could be used for microglial studies. Unfortunately, multiple popular and frequently used markers of microglia are not specific enough for reliable interpretation of research results. Nowadays, recognition of microglial spatial and temporal heterogeneity, the emerging new subtypes, and their complex and dynamically changing phenotypes require the use of either more adequate or multiple markers. Reasonable use of specific markers is essential for the progression of studies on glial functioning in physiology and disease. Since most CNS diseases involve immunological processes, recognition of their exact mechanisms is essential for developing their treatment and diagnosis. This review helps to systematize, describe, and understand different types of microglia markers in order to facilitate the use of relevant tools for further studies.

## Author Contributions

AJ and KK wrote the manuscript according to the research they performed. MP read, corrected, and edited the manuscript content and created all Tables and Schemes.

## Conflict of Interest

The authors declare that the research was conducted in the absence of any commercial or financial relationships that could be construed as a potential conflict of interest.
